# Primary Hyperparathyroidism With Brown Tumor of the Mandible Misdiagnosed as a Giant Cell Tumor: A Case Report

**DOI:** 10.7759/cureus.56153

**Published:** 2024-03-14

**Authors:** Asmara Hussain, Fatima Shahid, Nur Ul Ain

**Affiliations:** 1 Otolaryngology, Rawalpindi Medical University, Rawalpindi, PAK; 2 Plastic and Reconstructive Surgery Department, Rawalpindi Medical University, Rawalpindi, PAK

**Keywords:** hypercalcemia, chronic hyperparathyroidism, osteitis fibrosa cystica, masked primary hyperparathyroidism, adult primary hyperparathyroidism, mandible, osteolytic bone lesion, giant cell tumor, brown tumor, hyperparathyroidism

## Abstract

In this case study, we present an incidentally discovered giant cell granuloma, which, upon detailed investigation, led to an unexpected diagnosis. A 36-year-old woman exhibited a bone lesion in the right retromolar trigone area, initially suspected of being malignant. However, histopathological examination revealed a giant cell tumor of bone. Further biochemical profiling, including serum calcium, phosphorus, and parathyroid hormone (PTH) levels, showed elevated PTH and hypercalcemia, prompting consideration of primary hyperparathyroidism and the diagnosis of a brown tumor due to this condition. This case underscores the importance of considering brown tumors associated with primary hyperparathyroidism as a potential differential diagnosis in patients with lytic bone lesions.

## Introduction

After diabetes and thyroid dysfunction, primary hyperparathyroidism (PHPT) is the third most common endocrine condition. [1]. The prevalence of PHPT is estimated to be between 0.2 and 0.3% [2]. The presence of high serum calcium and low serum phosphorus values has traditionally been used to diagnose PHPT. However, it has been established that individuals with PHPT might have serum calcium values that are within normal limits [3]. About 1/3rd of HPT cases are discovered during routine biochemical profiles showing hypercalcemia in asymptomatic individuals. Chronic hyperparathyroidism causes osteitis fibrosa cystica (OFC), which is a skeletal disorder caused by the overproduction of parathyroid hormone and is characterized by sub-periosteal bone resorption, bony cyst formation, and brown tumors involving long bones [3]. Sub-periosteal resorptions in the fingers, skull, and long bones, as well as generalized osteopenia, are all symptoms of osteitis fibrosa cystica [4].

Brown tumor, a benign osteolytic disease, has many radiological and histological characteristics of a giant cell tumor of bone. Brown tumors are found to be present in 0.1% of individuals with PHPT and 1.5% to 1.7% of those with subsequent illnesses [5,6]. A brown tumor might be the initial symptom of hyperparathyroidism. Brown tumors are histologically composed of mononuclear stromal cells, multinucleated giant cells, and hemorrhagic infiltrates with occasional hemosiderin deposits (thus the brown hue) frequently present [7]. Brown tumors can develop in any bone [8]. When the same sort of lesion is seen in individuals who do not have PHPT, however, the differential diagnosis becomes more difficult.

A giant cell tumor (GCT) of the bone is a rare primary bone tumor that makes up about 5% of all primary bone tumors [9]. A giant cell tumor is a locally destructive tumor that is characterized by a large number of multinuclear giant cells with mature osteoclast-like characteristics [10]. The majority of people with GCT are asymptomatic or have bone discomfort as a result of tumor growth. GCT malignant transformation is an uncommon occurrence, occurring in less than 1% of patients [11]. Primary GCT is almost always treated surgically [9].

Biochemical analysis is used to make a differential diagnosis. Here, we report the case of a patient with an incidentally discovered giant cell granuloma, the diagnostic investigation of which led to an unexpected diagnosis.

## Case presentation

We report a case of a 36-year-old female presented in the outpatient department of Holy Family Hospital, Rawalpindi, with complaints of paresthesia and numbness of the right angle of the mandible for the past nine months and swelling involving the right angle of the mandible with bleeding gums for the past six months. There was a history of cold intolerance, fatigue, weight loss, and decreased appetite. The patient had no complaints of headache, cough, fever, dyspnea, dysphonia, or dysphasia. The patient has a history of lithotripsy in 2015.

Examination of the oral cavity revealed a firm, fleshy mass arising from the retromolar trigone and extending until the first lower molar tooth on the right side, which was non-tender and covered with mucus secretions. Oro-dental hygiene was satisfactory. The systemic examination was normal, with no such swelling noted in the rest of the body.

A soft tissue incisional biopsy of the right retromolar region revealed fibrillar connective tissue with spindly mononuclear cells mixed with uneven clusters of multinucleated giant cells. Prominent small capillary proliferations were also seen, which confirmed giant cell granuloma. Serum calcium was done, which was mildly elevated. PTH was elevated, and vitamin D levels were low. Baseline investigations were also done (Table 1, 2). Hence, a suspicion of hyperparathyroidism was raised, and a workup was done to confirm the diagnosis.

**Table 1:** Disease-specific investigations Sr: serum; TSH: thyroid stimulating hormone

Test	Results	Unit	Normal range
Sr calcium	12.0	Mg/dl	8.4-10.2
Intact parathyroid hormone	384	Pg/ml	15.0-65.0
TSH	4.70	uIU/ml	O.35-4.94
Vitamin D	18	nmol/L	75-250
Inorganic phosphorus	6.64	Mmol/l	0.81-1.62

**Table 2:** Baseline investigations WBCS: white blood cells

Test	Results	Unit	Normal range
Hemoglobin	9.8	g/dl	12-15g/dl
WBCS	11200	u/L	4,000-10,000
Platelets	6,90,000	u/L	1,50,000-4,10,000
Prothrombin time	13	sec	Control 11-16
Partial thromboplastin time	32	sec	26-40 sec

**Figure 1:**
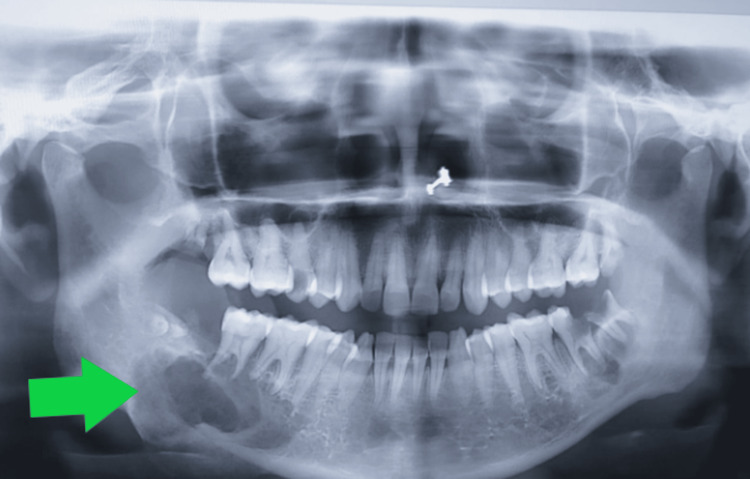
Giant cell granuloma in retromolar trigone

An ultrasound neck was performed, which showed mixed echoes of a soft tissue mass of 3.4 x 3.1 cm over the right mandibular bone. The left lobe of the thyroid is normal in size. An ovoid echopenic area of 14.4 x 6.7 mm is seen along the posterolateral aspect of the thyroid left lobe. An orthopantomogram X-ray was done, which depicted a lytic lesion in the left retromolar trigone region (Figure 1). A Technicium 99m sestamibi scan showed a cold nodule on the right lower lobe, negative for parathyroid adenoma. A bone scan was done, which showed active bone lesions in bilateral retromolar trigones and osteoarthritic changes in the dorso-lumber spine and shoulders. A presumptive diagnosis of parathyroid adenoma due to primary hyperparathyroidism was made.

**Figure 2:**
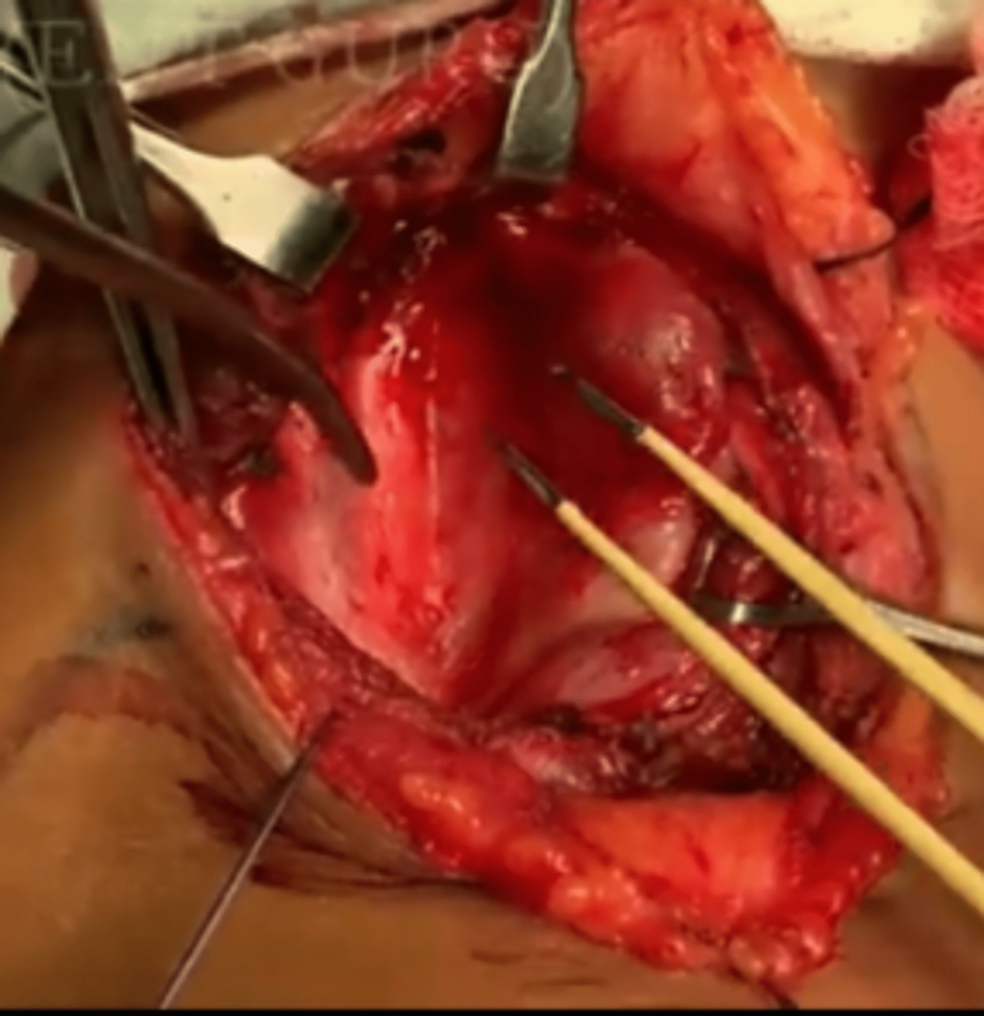
Pre-operative parathyroid adenoma Parathyroid adenoma located in the left lobe of the thyroid is indicated by monopolar cautery.

**Figure 3:**
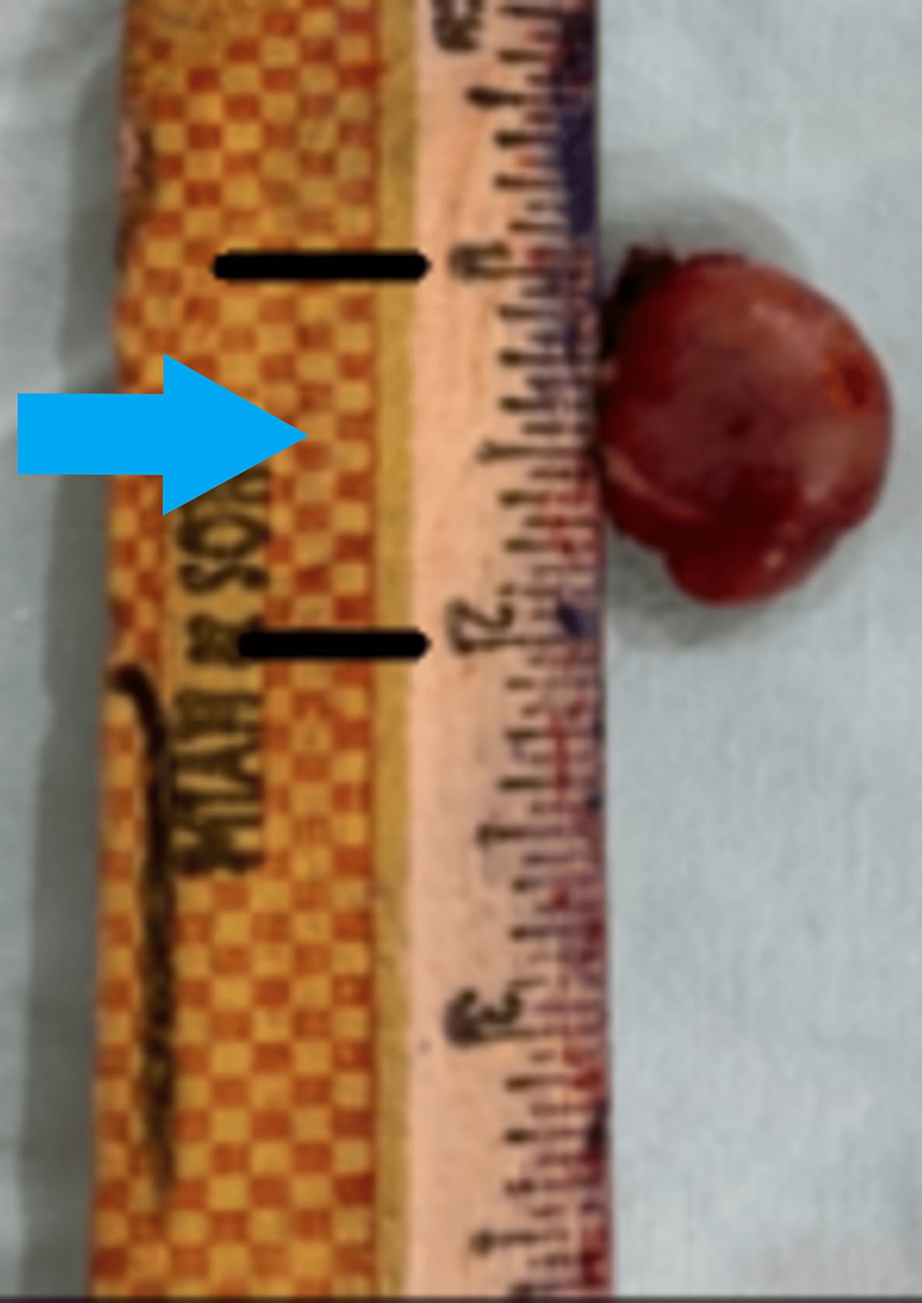
Post resection parathyroid adenoma

The patient was operated on under general anesthesia. A trans-cervical incision was made two finger breaths above the sternal notch. A left parathyroid adenoma measuring 1 x 2.5 cm was separated (Figures 2, 3). Left thyroid lobe removed. All vital structures were preserved during surgery. All cranial nerves were intact, and the specimen was sent for histopathology. The section revealed parathyroid tissue, showing an encapsulated lesion composed of nests and sheets of chief cells with round nuclei, indistinct nucleoli, and pale cytoplasm.

## Discussion

Brown tumors are associated with primary hyperparathyroidism and represent a reactive process due to excessive parathyroid hormone (PTH) secretion, while giant cell tumors are primary bone neoplasms. Brown tumors are a rare manifestation of primary hyperparathyroidism (PHPT), characterized by osteoclastic giant cell-rich lesions resulting from the excessive secretion of parathyroid hormone (PTH) [12]. While their occurrence is infrequent, brown tumors can affect various bones, including the mandible, ribs, and long bones [12,13]. Importantly, their clinical and radiographic features often mimic those of giant cell tumors or other lytic bone lesions, making differential diagnosis challenging. Misdiagnosis can lead to unwarranted surgical interventions or delayed treatment of the underlying hyperparathyroidism [12,13].

To differentiate brown tumors from giant cell tumors, clinicians must carefully evaluate the patient's clinical presentation, laboratory findings, and imaging results. Key diagnostic tools include the measurement of serum calcium and PTH levels, which are typically elevated in PHPT cases [13,14,15]. Additionally, dual-energy X-ray absorptiometry (DXA) scans are invaluable for assessing bone density and identifying characteristic changes associated with brown tumors [12]. The confusion between brown tumors and giant cell tumors can be attributed to the shared histological features, such as the presence of giant cells in both entities. However, their underlying causes, clinical courses, and treatments differ significantly. Therefore, accurate differentiation between the two is crucial for appropriate patient management and prognosis.

Furthermore, a comprehensive review of the medical literature reveals numerous documented cases where brown tumors were initially misdiagnosed as giant cell tumors, highlighting the diagnostic challenges and overlapping clinical presentations of these rare pathological entities in the realm of bone disorders. [13]. This underscores the significance of considering PHPT in the differential diagnosis of lytic bone lesions and emphasizes the importance of interdisciplinary collaboration between specialists, including endocrinologists, radiologists, and orthopedic surgeons, in such complex clinical scenarios [12].

Several case reports and clinical studies have shed light on these diagnostic challenges. For instance, a study by Zhong et al. [16] documented a case where a brown tumor in the left humerus was initially misdiagnosed as a giant cell tumor, emphasizing the importance of considering hyperparathyroidism as an underlying cause in patients with bone lesions. Additionally, a case report by Kamal et al. [17] discussed a case of a brown tumor, presenting numerous lytic lesions appearing in the right humerus and various other bones, that was initially mistaken for a giant cell tumor. Furthermore, Aghaghazvini et al. [18] reported a case of primary hyperparathyroidism, which was initially misdiagnosed as a giant cell bone tumor of the maxillary sinus; however, on further investigation, it was confirmed to be a brown tumor of the maxilla.

In summary, the distinction between brown tumors and giant cell tumors in bone pathology requires careful evaluation of clinical history, radiological findings, and histological characteristics. Misdiagnosis can have significant implications for patient care, making it essential for clinicians to be aware of these cases and consider hyperparathyroidism as a potential underlying cause when encountering bone lesions with giant cells in their practice.

## Conclusions

A radiologically proven case of giant cell tumor should be completed by measurement of serum calcium, phosphorus, and PTH levels to rule out brown tumors secondary to primary hyperparathyroidism. We present a case of a patient presenting with swelling of the retromolar trigone. Initial assessment showed it to be a case of a giant cell tumor, but further workup weighed more towards a brown tumor, and surgical exploration was done. It revealed a parathyroid adenoma, and hence it was removed. Later on, patients complained about numbness and swelling in the oral cavity, which improved with time. Hence, because of the similarity of a giant cell tumor with a brown tumor radiologically and histologically, one must always rule out parathyroidism in a patient who presents with swelling consistent with a giant cell tumor on investigations.
